# Dual orexin receptor antagonist induces changes in core body temperature in rats after exercise

**DOI:** 10.1038/s41598-019-54826-3

**Published:** 2019-12-05

**Authors:** Tristan Martin, Yves Dauvilliers, Ouma-Chandrou Koumar, Stéphane Besnard, François Dauphin, Nicolas Bessot

**Affiliations:** 1 Normandie Univ, Unicaen, INSERM, COMETE, 14000 Caen, France; 2 Reference National Center for Narcolepsy, Sleep Unit, Department of Neurology, Gui-de-Chauliac Hospital, University of Montpellier, Montpellier, INSERM U1061 France

**Keywords:** Peptides, Neurophysiology

## Abstract

Hypothalamic orexin neurons are involved in various physiological functions, including thermoregulation. The orexinergic system has been considered as a potent mediator of the exercise response. The present study describes how the antagonization of the orexinergic system by a dual orexin receptor antagonist (DORA) modifies the thermoregulatory process during exercise. Core Body Temperature (CBT) and Spontaneous Locomotor Activity (SLA) of 12 male Wistar rats were recorded after either oral administration of DORA (30 mg/kg or 60 mg/kg) or placebo solution, both at rest and in exercise conditions with treadmill running. DORA ingestion decreased SLA for 8 hours (*p* < 0.001) and CBT for 4 hours (*p* < 0.01). CBT (°C) response was independent of SLA. The CBT level decreased from the beginning to the end of exercise when orexin receptors were antagonized, with a dose-dependent response (39.09 ± 0.36 and 38.88 ± 0.28 for 30 and 60 mg/kg; *p* < 0.001) compared to *p*lacebo (39.29 ± 0.31; *p* < 0.001). CBT increased during exercise was also blunted after DORA administration, but without dose effects of DORA. In conclusion, our results favor the role of orexin in the thermoregulation under stress related to exercise conditions.

## Introduction

Core Body Temperature (CBT) is modulated by heat production and dissipation mechanisms controlled by the hypothalamus. This generates patterns of autonomic, endocrine, motor, and behavioral responses to adapt to environmental challenges^1^. Thermogenesis (heat gain) is mainly achieved through brown adipose tissue (BAT) sympathetic activation and shivering behavior whereas cutaneous vasoconstriction and evaporation regulate thermolysis (heat loss) (see review in^2^). CBT is regulated by a complex balance between feedback and feedforward mechanisms integrated at the level of hypothalamic preoptic area (POA)^3^. The lateral and posterior parts of the hypothalamus contain thermosensitive neurons synthetizing orexin/hypocretin, a neuropeptide mediated by two G-protein coupled receptors (OX_1_R and OX_2_R)^4,5^. Orexin neurons are distributed throughout the lateral hypothalamic area, but also in the dorsomedial hypothalamus and the medial preoptic area and projects to the medullary raphe one^6,7^. Moreover, orexin neurons receive inputs from the ventrolateral preoptic area, the locus coeruleus, the dorsal raphe, and the suprachiasmatic and tuberomammillary nuclei (see review in^8^). Their distribution infer a role of orexin in stress induced thermogenesis through glutamatergic excitatory neurotransmission from the dorsomedial hypothalamus to sympathetic premotor neurons in the rostral medullary raphe region^9^, resulting in BAT thermogenesis^10^. Experiments using orexin receptor antagonists^11^ have demonstrated an attenuated increase in CBT in response to stress. The orexinergic system has thus been hypothesized as a potent mediator of the exercise response. Related exercise increased in CBT is sensed by thermoreceptors located in the hypothalamus and periphery^12,13^ to produce appropriate thermoregulatory response. The orexinergic system may modulate the thermogenic response at central level with an increase of orexin levels in the cerebrospinal fluid in rodents and in dogs after a stressful condition^14,15^. As orexin receptors are also found in multiple peripheral mammal tissues^16,17^, the activation of the orexin system may also stimulate the autonomic nervous system in increasing the blood pressure to regulate heat balance^18^.

To our best knowledge, the link between the orexinergic system and thermoregulatory processes during exercise remains unclear. The stress-related thermal response during exercise may be modulated by different factors including the orexin system. Inactivation of both receptor subtypes (using pharmacological antagonization, surgery or in genetically modified animals) previously showed a decrease in CBT and spontaneous motor activity (SLA)^19–21^. We may thus hypothesize that thermoregulatory process linked to exercise would be modified by the inactivation of orexin receptors.

The aim of this study is to evaluate the effect of dual orexin receptor antagonist (DORA) on CBT. Measurements of CBT and SLA at rest and during exercise in rats under controlled conditions and after antagonizing the orexinergic system were used to assess this hypothesis.

## Results

### Effect of DORA 12 administration on SLA and CBT in resting conditions

**Figure 1:**
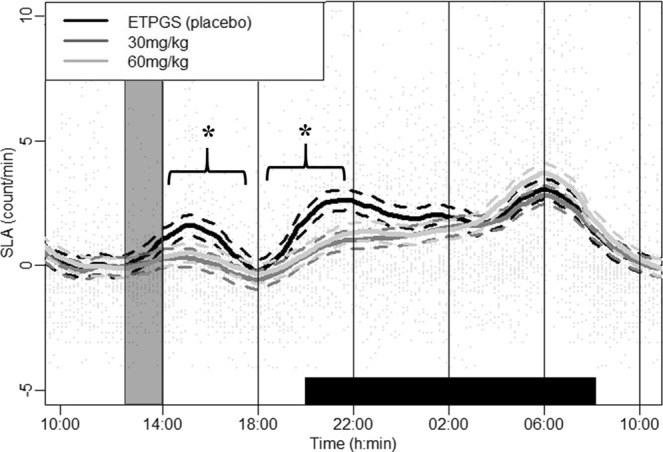
SLA profile (forced feeding between 13:00 and 14:00 represented by a shaded window) during the 24-hour free living condition before and after oral forced feeding of DORA 12 at 60 mg/kg (light gray line), 30 mg/kg (dark gray line), and placebo (black line) over time. Black bar on the x-axis represent the 12 h dark period. The thick line represents group smoothed curves obtained using polynomial regression (with span parameter α = 0.25). The dashed lines around the solid thick line correspond to a confidence band at level 100 × $\sqrt{0.95} \%$. Vertical lines separate the six 4-h periods used for mixed model analysis. *Difference between placebo and DORA 12, regardless of dose (p < 0.001).

All SLA recordings of rats during the 24-hour free living condition (without exercise) were reported before and after oral forced feeding of DORA 12 at 30 mg/kg, 60 mg/kg and placebo over time (Fig. 1). A mixed linear model comparison revealed a drug × period interaction (*p* < 0.001, Table 1). Post hoc analysis reported reduced SLA during the first 4-hour period after forced feeding for the 30 mg/kg (*p* < 0.001) and the 60 mg/kg (*p* = 0.02) DORA 12 dose when compared to the control condition. This first peak of SLA corresponds to a stress response to handling and oral forced feeding. Reduced SLA compared to the control condition was also observed during the second 4-hour period after forced feeding for 30 mg/kg (*p* < 0.001) and 60 mg/kg (*p* < 0.001) DORA 12 doses. This second peak of SLA corresponds to the beginning of the active phase in rats. The mean SLA level at this phase was thus blunted in rats receiving DORA 12. No SLA differences were found between the two DORA 12 doses tested in this study. Decreased SLA was observed during the 8 hours after antagonization of the orexinergic receptors with no dose effect.

**Table 1:** Model selection for testing dose and period effects on SLA during the 24-h free living condition.

Model		df	AIC	BIC	logLik	Test	L.Ratio	p-value
SLA = constant	1	3	24011.62	24031.02	−12002.81			
SLA ~ period	2	8	23754.74	23806.47	−11869.37	1 vs 2	266.88904	**<0**.**0001**
SLA ~ period * drug	3	20	23699.18	23828.51	−11829.59	2 vs 3	79.55741	**<0**.**0001**

For the description of table contents, see explanations in the data analysis and statistics section.

**Figure 2:**
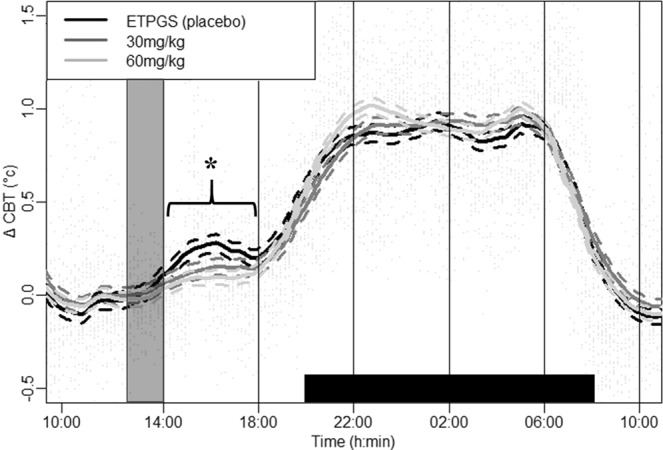
CBT profile (Δ CBT) (forced feeding between 13:00 and 14:00 represented by a shaded window) during the 24-h free living condition before and after oral forced feeding of DORA 12 at 60 mg/kg (light gray line), 30 mg/kg (dark gray line), and placebo (black line) over time. Black bar on the x-axis represent the 12 h dark period.The thick line represents group smoothed curves obtained using polynomial regression (with span parameter α = 0.25). The dashed lines around the solid thick line correspond to a confidence band at level 100 × $\sqrt{0.95} \%$. Vertical lines separate the six 4-h′ periods used for mixed model analysis. *Difference between placebo and DORA 12, regardless of dose (p < 0.001).

CBT measurements show a drug × period interaction (*p* < 0.001) (Table 2). As SLA should affect CBT, analysis was performed with and without SLA as a covariable in the model. A drug × period interaction was observed on CBT during the 24-h free-living condition, whether SLA was integrated into the model or not. A drug effect condition was observed only during the first 4-hour period (Fig. 2).

**Table 2:** Model selection for testing SLA, dose and period effects on CBT during the 24-h free living condition.

Model		df	AIC	BIC	logLik	Test	L.Ratio	p-value
CBT = constant	1	3	7033.768	7053.167	−3513.884			
CBT ~ period	2	8	4126.23	4177.958	−2055.114	1 vs 2	2917.54	<**0**.**0001**
CBT ~ dose * period	3	20	4097.83	4227.16	−2028.92	2 vs 3	52.392	<**0**.**0001**
CBT ~ dose * period + SLA	4	21	2710.25	2846.04	−1334.13	3 vs 4	1389.58	<**0**.**0001**

For the description of table contents, see explanations in the data analysis and statistics section.

Reduced CBT measurements compared to the placebo condition were observed during the first 4-hour period (14:00 to 18:00) after forced feeding for 30 mg/kg (*p* < 0.02) and 60 mg/kg (*p* < 0.001) DORA 12 doses. Post hoc analysis reported no differences in CBT between both DORA 12 doses tested. A non-dose-dependent decreased CBT was observed during the 4 hours after antagonization of the orexinergic receptors.

Fourty minutes after forced feeding, CBT increased by 0.04 °C ± 0.14 °C under placebo and changed by −0.04 ± 0.18 and 0.04 ± 0.14 °C after for 30 and 60 mg/kg DORA doses respectively. CBT reaches a maximum value 2 h post forced feeding in resting conditions. CBT had a mean maximal increase of 0.45 ± 0.40 °C after placebo administration. After DORA administration, CBT reached a maximum value of 0.22 ± 0.17 °C and 0.22 ± 0.15 °C for 30 and 60 mg/kg doses respectively.

### Effect of DORA 12 administration on SLA and CBT during exercise conditions

No dose-dependent changes in SLA were observed during stress-associated exercise (LR = 1.76; *p* = 0.41).

**Figure 3:**
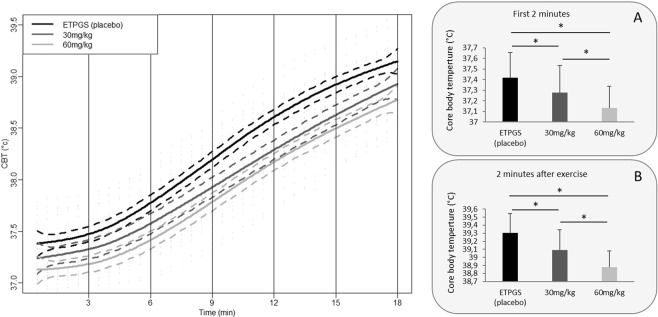
Profile of raw values of CBT, on the left. During the exercise bout after oral forced feeding of DORA 12 at 60 mg/kg (light gray line), 30 mg/kg (dark gray line), and placebo (black line) over time. The thick line represents group smoothed curves obtained using polynomial regression (with span parameter α = 0.25). The dashed lines around the solid thick line correspond to a confidence band at level 100 × $\sqrt{0.95} \%$. Histograms on the right indicates the mean temperature during the first 2 minutes before exercise (**A**) and during the 2 minutes after exercise (**B**). *Indicates a significant difference.

At the beginning of the exercise session (mean of the first 2 minutes of exercise), DORA 12 ingestion caused a drug-dependent reduction of the CBT level (LR = 73.22; *p* < 0.001). Mean CBT after 30 mg/kg and 60 mg/kg of DORA 12 was 0.15 °C (37.27 ± 0.25 °C; *p* < 0.001) and 0.27 °C (37.13 ± 0.20 °C; *p* < 0.001) lower than after placebo administration (37.42 ± 0.24 °C) respectively (Fig. 3A). CBT was also lower after 60 mg/kg than after 30 mg/kg of DORA 12 (*p* < 0.001).

At the end of exercise (mean of the 2 first minutes after exercise), DORA 12 ingestion caused a drug-dependent change (Fig. 3A) in CBT level (LR = 93.08; *p* < 0.001). CBT was still lower after intake of 60 mg/kg (38.88 ± 0.28; *p* < 0.001) or 30 mg/kg (39.09 ± 0.36 °C; *p* < 0.001) of DORA compared to placebo intake (39.30 ± 0.32). Differences between 30 mg/kg and 60 mg/kg doses was also significant at the end of exercise (*p* < 0.001). All results are displayed in Fig. 3.

At the end of exercise, CBT had a mean increase of 1.77 ± 0.29 °C after placebo administration. After DORA administration, temperature reached a maximum value of 1.69 ± 0.32 °C and 1.65 ± 0.32 °C for 30 and 60 mg/kg doses respectively.

**Figure 4:**
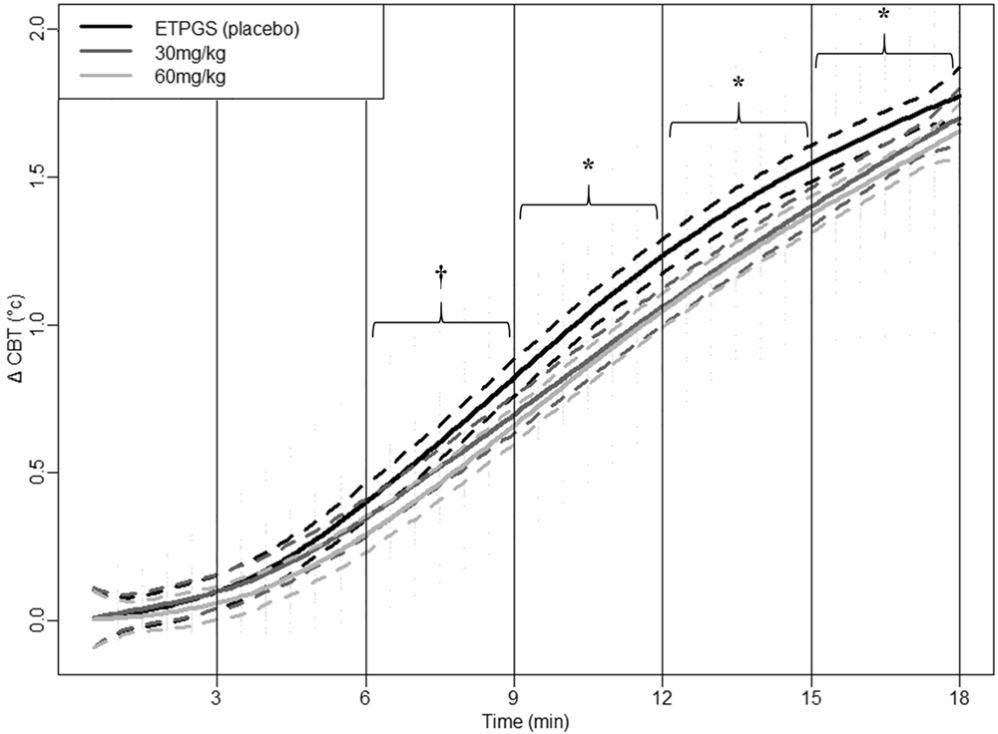
Δ CBT from the first minute before the exercise bout after oral forced feeding of DORA 12 at 60 mg/kg (light gray line), 30 mg/kg (dark gray line), and placebo (black line) over time. The thick line represents group smoothed curves obtained using polynomial regression (with span parameter α = 0.25). The dashed lines around the solid thick line correspond to a confidence band at level 100 × $\sqrt{0.95} \%$. Vertical lines separate the six 3-min periods used for mixed model analysis. ^†^Difference between DORA 12 at 60 mg/kg and placebo (p < 0.001). *Difference between placebo and DORA 12 regardless of dose (p < 0.001).

CBT kinetics during exercise are shown in Fig. 4. When data were subtracted at beginning of exercise, (considered as 0) to unmask the effect of DORA on CBT described above, the mixed model analysis revealed that Δ CBT kinetics are significantly modified by DORA 12 ingestion (Table 3). After 9 minutes of exercise, Δ CBT shows a sharper increase after placebo ingestion than after both DORA 12 administrations (*p* < 0.001) regardless of DORA 12 concentration. Moreover, a significantly lower increase in CBT is observed between the 6th and 9th minute after DORA 12 administration with 60 mg/kg compared to placebo (p < 0.001).

**Table 3:** Model selection for testing dose and period effects on CBT during exercise.

Model		df	AIC	BIC	logLik	Test	L.Ratio	p-value
CBT = constant	1	3	2094.41	2109.57	−1044.21			
CBT ~ period	2	4	−354.12	−333.92	181.06	1 vs 2	2450.54	<**0**.**0001**
CBT ~ dose * period	3	8	−444.73	−404.33	230.36	2 vs 3	98.61	<**0**.**0001**

For the description of table contents, see explanations given in the data analysis and statistics section.

## Discussion

We assessed the involvement of the orexinergic system on the SLA level and CBT response at rest and under a stress associated exercise response in rats. Antagonization of the orexinergic system by DORA ingestion induced decreased SLA for 8 hours and decreased CBT for 4 hours in resting conditions, and a blunted CBT increase during exercise.

The orexinergic system promotes wakefulness and excitability, with high levels of orexin in the brain during the waking period and low levels during the sleep period^22–25^. This property implies a major role of orexin in the regulation of the rest-activity rhythm^26,27^. In the present study, the 30 mg/kg of DORA 12 dose used should have resulted in an inactivation of 97% of orexin receptor in the brain^28^, logically impairing the orexin wakefulness-promoting activity.

DORA caused a significant change in rat behavior. First, the amount of stress-related SLA was lower in rats receiving DORA 12 solution, leading to less movement during the first 8 hours. Then, the amount of SLA was also blunted during the first part of their habitual activity phase after DORA 12 administration, with no dose effect. Results from the present study were in accordance with previous studies in which DORA promoted sleep behavior and reduce SLA for several hours^29–31^ and blocked the arousal-promoting activity of orexin peptides, with higher efficacy during the active phase in rats^28^.

Without exercise, all rats first displayed a lower mean CBT level up to 4 h after DORA 12 ingestion compared to E-TPGS placebo solution. Under normal conditions, CBT is regulated through heat gain and heat loss mechanisms to maintain the CBT close to a “set point” specific to each species^32^. However, throughout the 24 h period, the endogenous mechanism driven by the circadian clock in the suprachiasmatic nucleus will modulate this mechanism to allow a CBT rise during the active phase and a decrease during the resting phase^33,34^. During the resting phase, thermoregulation mechanisms promote an increase in the distal cutaneous temperature, leading to a decrease CBT. Redirection of the blood flow to the body’s extremities (hands and feet) allows heat transfer towards the environment (see review in^35^). A reverse situation occurs during the active phase. In reducing CBT, we may speculate that DORA ingestion may promote heat loss mechanisms by increasing blood flow to the body’s extremities, reduce heat gain mechanisms by inducing changes in thermogenesis, or both.

The present study found persistence of DORA effects on CBT whether SLA was added as a covariable in the analysis model or not, meaning that CBT changes were independent of SLA. Such an independence between SLA and CBT was earlier observed after anesthesia in orexin neuron-ablated mice (ORX-AB), with a delay in the recovery of body temperature and locomotor activity after withdrawal of anesthesia^36^. The “resting condition” (forced feeding with no following exercise) was set to control the effect of DORA’s compound effectiveness. Exercise or rest condition were counterbalanced to avoid a possible habituation. The effect of orexin system disruption on response to stress has been previously described^20,37^ as described below. However, it was indispensable to control DORA’s effect at rest to control the effect of DORA 12 compound before any subsequent exercise. Orexin neuron-ablated mice did not tolerate 4 h of cold exposure compared to wild type mice, but had a similar locomotor activity response^37^. The present study confirmed that thermoregulation alteration in orexin deficient animals is not due to changes in locomotor activity.

Rats in this study were left to rest after DORA 12 administration because handling and oral forced feeding caused a significant amount of stress in the animals despite previous habituation. This is reflected in the sudden hyperthermia and increased SLA following oral forced feeding. The CBT response was blunted after DORA 12 ingestion and led to a lower sustained level of CBT for 4 hours. A blunted hyperthermia response to acute stressors has also been observed in orexin neuron-ablated mice after induced stress by handling^20^. These authors observed with a very slow and gradual increase (0.4◦ C) in CBT, with a peak reached 70 min after handling, while a normal temperature change in response to handling stress was found in both wild type and prepro-orexin knockout mice (ORX-KO). Altogether, these results suggest that attenuated stress-induced hyperthermia relates to loss of orexin neurons rather than the orexin peptide itself, thus involving other neurotransmitters^8,20^.

During exercise, heat produced by exercising muscles is counteracted by an increase in heat loss mechanisms to maintain the thermal balance^38^.

The preoptic area and the hypothalamus are important mediators of thermoregulation in response to exercise. By infusing tetrodotoxin to inhibit neurotransmission in these brain regions^39^, impairment of heat loss and enhancement of heat production were found during exercise, suggesting that the preoptic area and the hypothalamus are important areas for heat loss as opposed to heat production during exercise. As orexin neurons are located in these areas, this system could be a potential candidate for mediating the thermoregulatory response to exercise.

Despite a slight stress-associated increase in CBT before exercise, rats in the present study started from a lower level of CBT after DORA 12 ingestion compared to 20% E-TPGS solution. Δ CBT rose at a lower rate after 18-minute bout of exercise with DORA intake, with a delay in increased CBT after DORA 12 ingestion compared to placebo. Exercise session caused a mean increase up to 3.9 higher than at rest with placebo and up to 7.6 times higher than at rest under DORA. Our results illustrated that lower CBT level after DORA ingestion did not influence the rise of CBT during a stress-associated exercise. All these results support the role of the orexin system in the CBT response to physical stressors and physiological defense responses. Indeed, stress from handling induced blunted hyperthermia responses in rats after administration of an OX_1_R antagonist^11^ as well as in orexin neuron-ablated mice^20^. Thermoregulatory responses to cold exposure are attenuated in orexin neuron-ablated rats^40^, whereas administration of orexin improved hypothermia and survival rate in mice submitted to septic shock after endotoxin injection^41^. Herein, we cannot totally assume that DORA administration “blunted” the exercise-induced hyperthermia. If exercise duration would have been extended, CBT might have plateaued to similar levels than placebo when heat balance was achieved. In this scenario, DORA ingestion would be more associated with a delayed onset in the rise in CBT during exercise.

The mechanisms by which inhibition of orexin receptors decrease the CBT exercise-related response remain however unclear. One hypothesis is that orexin receptor activation can induce thermogenesis through the activation of brown adipose tissue (BAT) sympathetic nerves from the raphe pallidus^10,42–45^. Under acute stress or cold, orexin neurons activate the dorsomedial hypothalamic nucleus which acts as a hub for stress signaling, with monosynaptic projections to the raphe pallidus for sympathetic outputs that drives BAT thermogenesis^6,9,46,47^. At the reverse, the destruction of orexin neurons in mice induced abnormal BAT thermogenesis. BAT thermogenesis is not dependent on the SLA level, suggesting a role of BAT in non-shivering thermogenesis, at least in animals^48^. Stress-induced thermogenesis would likely be due to BAT thermogenesis rather than prevention of heat loss by cutaneous vasoconstriction^20^. The precise role of exercise in BAT activity remains unclear^49^; however recent data favors that chronic exercise is a weak stimulus for BAT thermogenesis^50^. In contrast, a one-hour exercise bout increased BAT in humans^51^. The involvement of either orexin receptor 1 or 2 was not assessed in this study; however the orexin signal and its receptor 1 have a key role for thermogenic BAT effects^52^. Altogether, we may hypothesize that antagonization of orexin 1 receptor signaling by DORA administration in the present study could lower BAT thermogenesis and account for the lower increase in CBT during exercise. We may also suggest that after DORA ingestion, orexin release during exercise cannot exert its wakefulness-promoting role on targeted brain areas. Indeed, orexin neuron activation rates have been linked to behavior, with increased vigilance (e.g. exploration) and also have a strong emotional component^24^. Lowering the behavioral level may also participate in the global impaired thermogenesis after orexin receptor antagonization.

All these findings may suggest challenging these results in patients suffering from orexin-deficient type 1 narcolepsy. Type 1 narcolepsy is an orphan disorder characterized by excessive daytime sleepiness, cataplexy, disturbed nighttime sleep, sleep paralysis, and hypnagogic hallucinations^53,54^. Previous studies have reported modifications in heat loss, with lower proximal and central temperature and higher distal temperature in narcoleptic patients^55–59^. A recent small study reported similar levels of sympathetic and metabolic activity of BAT in patients with narcolepsy and controls, suggesting normal non-shivering thermoregulation in narcolepsy despite the lack of orexin neurons^58^. To explain such discrepancies between human and animal models of narcolepsy, further studies are required to better understand the thermoregulatory mechanisms, vasoregulation, sympathetic innervation, and metabolic activity of BAT as a function of orexin activity.

The present study has some limitations. First, only SLA was recorded to examine the effect of orexin receptor antagonization; however no sleep recording was performed for this study that precludes to report any sleepiness DORA effect. Thus, it was not possible to precisely determine how DORA 12 administration changes the sleep-wake cycle in rats during our experiment. Second, although DORA 12 influences the CBT during exercise, our study cannot elucidate whether and how it affects the thermoregulatory centers or other factors such as sleeping time, a reduction in muscle tone, on general metabolism and vasoregulation. Third, the present experimental design involved a stress response to oral gavage before exercise session. Future studies should consider DORA delivering via indwelling gastric cannula to avoid handling. However, exercise caused a mean increase up to 3.9 higher than at rest with placebo and up to 7.6 times higher than at rest under DORA. Even though our results does not reflect a “pure” exercise answer, the magnitude of the increase of temperature at exercise compared to the oral gavage induces-stress alone, cannot be ignored or only confounded with stress. Fourth, we administered DORA 12 at only one time of day, during the heat gain phase. This was based on personal data showing that the effect of exercise was more pronounced during the heat gain phase than during the heat loss phase^60^. However, it would have been useful to test potential differences between exercise performed during heat gain and heat loss phases after DORA 12 administration. Fifth, we did not measure the BAT thermogenesis. Further studies should include BAT thermogenesis recording to better understand whether orexin system antagonization influences BAT thermoregulation or only the cutaneous vascular beds. Finally, we did not record tail temperature which would have enabled us to observe how the antagonization of the orexinergic system influences cutaneous vasomotion.

In conclusion, our present study showed that orexin receptor antagonization decreased the rise in CBT in both resting and exercise conditions. The blunted hyperthermia-related response to handling and oral forced feeding and the sustained lower CBT level lasting for hours after DORA 12 ingestion support the hypothesis that orexin is key in in the thermoregulation response to stressful events. This study expands on our knowledge of the role of orexin in the thermoregulation under stress conditions related to exercise conditions.

## Methods

### Ethical approval

Experiments were carried out in accordance with the European Communities Council Directive 2010/63/UE and French law. The protocol was approved by the animal ethical committee: Comité National de Réflexion Ethique sur l’Expérimentation Animale (CENOXEMA- 05087.01).

### Animals

Twelve male Wistar rats (350–500 g, Janvier, France) were individually housed under constant temperature (23 ± 1 °C) and (hygrometry; 50 ± 5%) with 12:12-h light-dark cycle (light from 8:00 to 20:00). Environmental conditions were kept as constant as possible during all the experiment. Food and water were available ad libitum.

### General procedure

Rats were housed in a box dedicated to the experiment and were regularly handled and familiarized with oral forced feeding to avoid stress during the final experiment. One week before the experiment, each rat was implanted with a telemetric device (TA F40, Data Sciences International, St. Paul, MN) to continuously measure CBT and SLA. After an 8-day convalescence period, rats were habituated to treadmill running before the beginning of the testing sessions.

Rats received successively, in a counterbalanced order, an oral administration of placebo, 30 mg/kg of DORA-12 solution, and 60 mg/kg of DORA-12 solution. Each rat received each solution twice, once in resting condition and again in exercise condition. Each of the 6 oral administration sessions were separated by a period of at least 72 hours. The resting condition (forced feeding with no following exercise) was set to control the effect of DORA’s compound effectiveness on SLA and CBT. Exercise conditions consisted of 18-minute exercises on a running treadmill (1 minute at 15 cm.s^−1^, 30 s of acceleration up to 30 cm.s^−1^, followed by 16.5 min at 30 cm.s^−1^).

Each experimental session occurred during the heat gain phase of the animal, between 15:00 and 17:00. When rats were submitted to the exercise conditions, the oral administration took place between 40 and 45 minutes before. CBT and SLA were recorded 5 hours before oral forced feeding and continued during the night after the test session for 20 h. The first hour was not included in the analysis to avoid artefacts due to cage and rat manipulation.

### Dual antagonist orexin receptor

Rats were subjected to oral administration of 30 mg/kg and 60 mg/kg of DORA-12 diluted in a 20% vitamin E TPGS solution (D-α -Tocopheryl polyethylene glycol 1000 succinate) (Sigma Aldrich^®^). DORA-12 compound was provided by Merck & Co., Inc. (2000 Galloping Hill Road, Kenilworth New Jersey, USA 07033). Previous studies reported that a 30 mg/KG oral administration was sufficient to achieve OX_1_ R and OX_2_ R occupancies of 97% and somnolence for up to six hours^28^. Placebo solution consisted of a vehicle solution (20% vitamin E-TPGS) without DORA-12.

The 20% vitamin E TPGS solution was prepared by heating the appropriate contents of TPGS to a liquid state (40–50 °C). Liquid E-TPGS was slowly poured into water heated to 60°–90 °C with vigorous stirring. After stirring the solution at moderate speed at room temperature for approximately 2 hours to ensure complete dissolution of the particles. The DORA 12 solution was prepared by adding the appropriate dose of DORA-12 according to the animal’s weight to the 20% E-TPGS and stirring again the solution at moderate speed at room temperature for 4 hours.

### Familiarization procedure with treadmill exercise

Rats were habituated twice a day to a forced exercise bout for 6 days on a rodent treadmill (Bioseb Co. Vitrolles, France). Forced exercise intensity was constant throughout the entire protocol (30 cm.s^−1^). Exercise duration gradually increased from 2 to 9 minutes. Among the 12 rats initially trained during the familiarization session, 11 were selected based on their ability to run without the need for negative reinforcement (0.5 mV electric foot shock).

### Recording of CBT and SLA

A TA F40 device (Data Sciences International, St. Paul, MN) was used to measure CBT and SLA, the latter being defined as all horizontal linear motions. Implantation of the sensor was carried out as described by the Data Sciences International requirements (see^61^). The device is a cylindrical implant inserted intra-abdominally after midline laparotomy under isoflurane anesthesia. Animals were kept in convalescence for 7 days after implantation of a telemetric sensor. An intraperitoneal injection of 2 mL of antibiotic (amoxicillin-clavulanic acid) and 2 mL of analgesic (paracetamol) was given once a day for 3 days to prevent nociception. CBT (°C) and SLA (count.min^−1^) values were collected at a sampling frequency of 30 s. All data were collected continuously during the recording phase. For the 24-h resting condition analysis, data were averaged by 10-min periods. In this condition, CBT measurements were subtracted before forced feeding between 13:00 and 14:00 (Δ CBT). In exercise conditions, for kinetic analysis CBT measurements were subtracted (Δ CBT) from the first minute before exercise (Temperature = 0 °C).

### Data analysis and statistics

Statistical analyses were carried out using R 3.1.2 software (www.r-project.org).

A mixed linear model (lme) analysis was selected to analyze DORA12 effects on CBT and SLA changes during the 24-h resting condition, and CBT during exercise. The resting period was divided into 6 periods of 4 hours, the first corresponding to the pre-ingestion period.

The exercise period was divided into 6 bouts of 3 minutes. We used the Akaike information criterion (AIC) and the Bayesian Information Criterion (BIC) as indicators for the most appropriate correlation structure. The typical time series autoregressive (AR(1)) form was selected.

To assess whether a variable had a significant effect, the approach of Pinheiro & Bates^62^ was followed, and models with and without the respective variable were compared by means of a likelihood ratio test (LRT). For analyses with several factors, these were included stepwise into the model. This started with the factor most likely to exert the strongest effect. Then, successive additions were made of those with a weaker effect (the factors used were periods (time cutting) and drugs (60 mg/kg of DORA 12, 30 mg/kg of DORA 12, and placebo). Tables show all models fitted to the data. The models are ordered by increasing complexity, starting with the “null model” consisting of a constant only. The columns show (from left to right) the model, the number of degrees of freedom (df) of the model, the Akaike information criterion (AIC), the Bayesian information criterion (BIC), the log-likelihood (logLik), the models tested by a Likelihood Ratio Test (LRT), the statistics of the LRT, and the *p*-value of the LRT. Table is interpreted as follows. Lower values of AIC and BIC indicate the best model. The LRT tests all successive pairs of models one after the other.

If any interaction effect was detected, Tukey’s significant difference test was used for post-hoc analysis. The level of statistical significance was set at *p* < 0.05 throughout the study.

The datasets generated during and/or analysed during the current study are available from the corresponding author on reasonable request.
