# Author Correction: Dual orexin receptor antagonist induces changes in core body temperature in rats after exercise

**DOI:** 10.1038/s41598-020-62685-6

**Published:** 2020-04-02

**Authors:** Tristan Martin, Yves Dauvilliers, Ouma-Chandrou Koumar, Valentine Bouet, Thomas Freret, Stéphane Besnard, François Dauphin, Nicolas Bessot

**Affiliations:** 1 Normandie Univ, Unicaen, INSERM, COMETE, 14000 Caen, France; 2 Reference National Center for Narcolepsy, Sleep Unit, Department of Neurology, Gui-de-Chauliac Hospital, University of Montpellier, Montpellier, INSERM U1061 France

Correction to: *Scientific Reports* 10.1038/s41598-019-54826-3, published online 05 December 2019

Valentine Bouet and Thomas Freret were omitted from the author list in the original version of this Article. This has been corrected in the PDF and HTML versions of the Article.

The Author Contributions section now reads:

Y.D., O.-C.K. and N.B established theoretical framework. V.B and T.F. helped with project administration and training of the experimenters. O.-C.K., V.B., T.F., and F.D. established and implemented the methodology. T.M. collected the data. T.M., Y.D., and N.B. analysed the data. N.B. performed statistical analysis. S.B. provided equipment. T.M., Y.D., S.B., F.D, and N.B. wrote the article.

